# Spatial distribution and driving factors of scalding tobacco risk in K326 flue-cured tobacco in Yunnan Province, China

**DOI:** 10.3389/fpls.2026.1922535

**Published:** 2026-09-22

**Authors:** Zhimei Yang, Changhui Xu, Khanom Simarani, Guoliang Li, Yukai Huang, Youshou Gao, Dongfang Zheng, Yonglei Jiang, Yi Chen, Yuwen Shao

**Affiliations:** 1 Yunnan Academy of Tobacco Agricultural Sciences, Kunming, Yunnan, China; 2 Division of Microbiology, Institute of Biological Sciences, Faculty of Science, Universiti Malaya, Kuala Lumpur, Malaysia; 3 Faculty of Civil Engineering and Mechanics, Kunming University of Science and Technology, Kunming, Yunnan, China; 4 Solar Energy Research Institute, Yunnan Normal University, Kunming, Yunnan, China; 5 Chuxiong Tobacco Bureau of Yunnan Province, Chuxiong, Yunnan, China

**Keywords:** curing-management measures, field management practices, MaxEnt model, meteorological factors, scalding tobacco

## Abstract

**Introduction:**

Scalding tobacco is a major curing-related defect that reduces the quality of flue-cured tobacco leaves. Its occurrence and severity are associated with meteorological conditions, field-management practices, and curing-management measures, but their relative roles and spatial patterns remain unclear. This study investigated the spatial distribution and major factors associated with scalding tobacco risk in K326 flue-cured tobacco across the major tobacco-growing regions of Yunnan Province, China.

**Methods:**

Meteorological data from 80 weather stations were combined with 3,106 field survey records from ten major tobacco-growing regions. Random Forest (RF), Shapley Additive Explanations (SHAP), and occurrence-risk models were used to evaluate severity-related associations and spatial risk patterns.

**Results:**

Meteorological variables showed limited predictive ability for scalding severity but contributed to spatial variation in occurrence risk. Annual sunshine duration was the most important meteorological predictor in the MaxEnt model, contributing 48.7%, and higher predicted risk was concentrated mainly in parts of central and western Yunnan. Field-management practices showed stronger associations with severity differentiation, with days after transplanting emerging as the most influential predictor. Among curing-management measures, curing protocol showed greater importance for spatial occurrence-risk prediction, whereas barn loading density showed a stronger association with severity variation.

**Conclusions:**

Scalding tobacco risk in K326 flue-cured tobacco was associated with regional meteorological conditions and field- and curing-management practices. These findings support a shift from curing-centered control toward integrated management combining meteorological risk identification, appropriate harvest timing, coordinated nutrient management, and precision curing. Further multi-year, multi-region, and multi-cultivar validation is needed to improve the generalizability of these findings.

## 1 Introduction

Flue-cured tobacco (*Nicotiana tabacum* L.) is one of the most important economic crops in China, contributing substantially to agricultural productivity, farmers’ income, and regional economic development (Xu et al., 2008; Wang et al., 2024a). However, the frequent occurrence of curing-related defects remains a major challenge to tobacco quality improvement and the sustainable development of the tobacco industry. Among these defects, scalding tobacco, characterized by localized or widespread grayish-brown discoloration on cured leaves, accounts for approximately 40–50% of all defective tobacco leaves and substantially reduces their industrial usability and market value (Wang et al., 2018; Chen et al., 2019). Previous studies have shown that the occurrence of scalding tobacco is jointly influenced by environmental conditions, agronomic management practices, and curing-process control (Yang, 2014).Yunnan Province, the largest producer of high-quality flue-cured tobacco in China, is characterized by a low-latitude plateau landscape with pronounced elevational gradients and diverse climatic conditions. Significant spatial variations in temperature, precipitation, solar radiation, and production management practices occur across tobacco-growing regions, resulting in marked spatial heterogeneity in the occurrence of scalding tobacco (Chen et al., 2023). Recent studies have further demonstrated pronounced regional differences in climatic conditions across the tobacco-growing areas of Yunnan Province and highlighted the important roles of temperature, precipitation, and sunshine duration in determining tobacco-growing suitability and leaf quality (Dou et al., 2024; Sun et al., 2025). Therefore, elucidating the spatial distribution patterns and underlying drivers of scalding tobacco risk across Yunnan Province is crucial for improving tobacco leaf quality, optimizing production management, and supporting the sustainable development of the tobacco industry.

In recent years, geographic information systems, remote sensing technologies, multi-criteria decision-making approaches, and machine-learning algorithms have been widely employed in agricultural ecological risk assessment and crop suitability analysis (Wang et al., 2024b). Among these approaches, the Maximum Entropy (MaxEnt) model has gained considerable attention because of its ability to establish robust relationships between target occurrences and environmental variables using limited sample data. Owing to its strong predictive performance and stability in handling imbalanced datasets and complex environmental conditions, MaxEnt has been extensively applied in crop suitability mapping, disease risk assessment, and agroecological evaluation (Wei et al., 2018; Xin et al., 2018). Recent studies further demonstrate its utility for identifying key environmental constraints and characterizing spatial variation in agricultural suitability and risk. For example, Ali et al. (2023) applied MaxEnt to assess the current and future potential cultivation suitability of wheat, soybean, and rice in East Asia, identifying precipitation- and temperature-related bioclimatic variables as important determinants of crop suitability. Ma et al. (2024) further used MaxEnt to predict the potential occurrence of major maize diseases under climate-change scenarios and identified temperature and precipitation as major climatic constraints on disease occurrence. Jia et al. (2024) assessed the global potential suitability of tobacco and identified climatic and edaphic factors as major determinants of its distribution, with the mean temperature of the warmest quarter (bio10) and annual precipitation (bio12) exhibiting particularly strong influences. Tagyan et al. (2025b) predicted the potential global distribution of Ralstonia solanacearum, the causal agent of tobacco bacterial wilt, and highlighted annual mean temperature as the primary environmental driver. Similarly, Tagyan et al. (2025a) employed MaxEnt to evaluate the global distribution of Fusarium proliferatum and its associated mycotoxin risk under climate change scenarios, identifying temperature seasonality as the most influential limiting factor. In addition, a growing number of studies have integrated remote sensing techniques, analytic hierarchy processes, and machine-learning models to assess crop suitability and agricultural risk at regional scales (Zhang et al., 2022; Tian et al., 2024; Chen et al., 2025). Collectively, these studies demonstrate the reliability and applicability of MaxEnt for agricultural spatial prediction and ecological risk assessment. Despite these advances, research on scalding tobacco has primarily focused on curing-process optimization, physiological and biochemical responses of tobacco leaves, and the effects of individual environmental factors on tobacco quality (He et al., 2007; Chen et al., 2019; Zhang et al., 2023). In contrast, the spatial distribution patterns of scalding tobacco risk and the interactions among multiple driving factors remain poorly understood. Methodologically, previous studies have rarely combined spatial occurrence-risk modeling with machine-learning interpretation of severity to distinguish the roles of climatic, field-management, and curing-management factors. At the regional scale, integrated assessments combining climatic observations with questionnaire-derived production-management information are also limited. These gaps constrain our understanding of the spatial heterogeneity and stage-specific drivers of scalding tobacco risk.

Therefore, this study investigated ten major tobacco-growing regions across Yunnan Province, China. By integrating meteorological observations from 80 weather stations with field management and curing-management information obtained from 3,106 questionnaire survey records, we combined Random Forest–SHAP (RF-SHAP) analysis with Maximum Entropy (MaxEnt) modeling to evaluate scalding tobacco risk from complementary severity and spatial perspectives. Specifically, we addressed three questions: (1) How do climatic conditions shape the regional spatial pattern of scalding tobacco risk across the major tobacco-growing regions of Yunnan Province? (2) Which field-management practices are most strongly associated with the differentiation of scalding tobacco severity? (3) How are curing-management practices associated with scalding tobacco occurrence and severity? By addressing these questions, this study aims to clarify the distinct roles of regional climatic conditions, pre-harvest field management, and post-harvest curing management in scalding tobacco risk across the major tobacco-growing regions of Yunnan Province, thereby providing a data-driven basis for integrated management combining climatic risk identification, appropriate harvest timing, coordinated nutrient management, and precision curing, and ultimately supporting sustainable tobacco production.

## 2 Materials and methods

### 2.1 Study area and data sources

This study was conducted during the 2025 tobacco-growing and curing season in the major flue-cured tobacco-growing regions of Yunnan Province, southwestern China. Yunnan Province is located between 97°31′–106°11′ E and 21°08′–29°15′ N and is characterized by a subtropical monsoon climate with localized tropical monsoon influences and pronounced vertical climatic variation. The province has an annual mean temperature of 17.5 °C, an average annual sunshine duration of 2,200 h, and an average annual precipitation of 1,100 mm. Owing to its diverse climatic and ecological conditions, Yunnan is recognized as one of the most important high-quality flue-cured tobacco-producing regions in China. To investigate the spatial distribution patterns and driving factors of scalding tobacco risk, field questionnaire surveys were conducted across ten major tobacco-growing regions of Yunnan Province (**Supplementary Table S1**). All survey records included in the study were obtained from K326 flue-cured tobacco. Information collected from each survey included geographic location, occurrence of scalding tobacco, tobacco cultivar, field management practices, and curing-management measures. The survey encompassed diverse ecological conditions and production-management systems within the major tobacco-growing regions, resulting in a total of 3,106 valid records. These data were used to establish the occurrence database of scalding tobacco and the associated risk-factor database. Meteorological data were obtained from the China Meteorological Administration and included annual precipitation (prec), annual mean temperature (temp), and annual sunshine duration (sun) recorded at 80 meteorological stations distributed across the ten tobacco-growing regions. The spatial distributions of meteorological stations and survey locations are shown in **Figure 1**. These datasets provided the basis for environmental variable construction, spatial analysis, and subsequent MaxEnt modeling.

**Figure 1:**
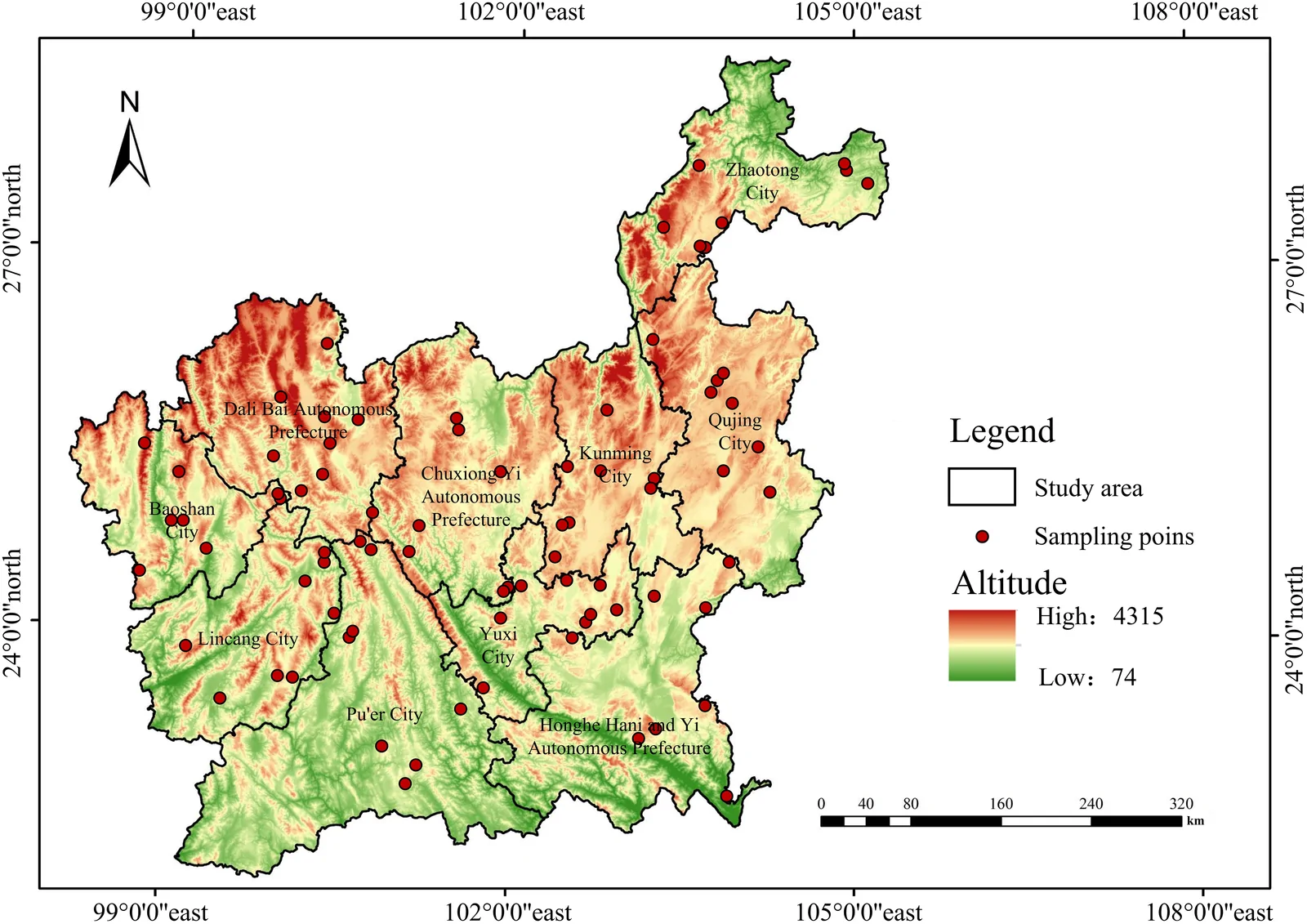
Distribution of tobacco leaf sampling points.

### 2.2 Variable classification and data preprocessing

Based on the formation process of scalding tobacco and the corresponding production stages, candidate explanatory variables were classified into three categories: meteorological factors, field management practices, and curing-management measures. Meteorological factors included precipitation, annual mean temperature, and annual sunshine duration, representing the regional environmental conditions associated with scalding tobacco occurrence. Field management practices included microbial inoculant application, potassium fertilizer management, potassium fertilizer type, potassium fertilizer application rate, days after transplanting, and harvest maturity, reflecting differences in nutrient management, leaf development, and harvest timing before curing. Curing-management measures included curing protocols and barn loading density, representing key management practices during the post-harvest curing process. Among these variables, precipitation, annual mean temperature, annual sunshine duration, potassium fertilizer application rate, days after transplanting, and barn loading density were treated as continuous variables. Microbial inoculant application, potassium fertilizer management, potassium fertilizer type, harvest maturity, and curing protocol were treated as categorical variables. Harvest maturity was recorded from farmer questionnaire responses and classified as early mature, mature, or over-mature; thus, it represents grower-assessed maturity rather than an objectively measured physiological maturity index.

To ensure data quality and consistency, all questionnaire records were subjected to standardized preprocessing procedures, including text standardization, missing-value identification, category harmonization, and numerical conversion of continuous variables. For descriptive analyses, continuous variables were categorized using predefined intervals or quartile-based groups, as appropriate, whereas their original continuous values were retained for subsequent model fitting. Categorical variables were standardized by merging synonymous or inconsistently labeled categories and were subsequently encoded for model input. Accordingly, continuous predictors were entered into the Random Forest and MaxEnt models in numeric form, while categorical predictors were entered according to their standardized categories. After data cleaning, a total of 3,106 valid survey records were retained. All variables contained 3,106 valid observations except for curing protocol, for which 302 records were missing. Consequently, analyses involving curing protocol were conducted using 2,804 complete records. To evaluate the potential influence of these missing records, a sensitivity analysis was additionally performed by assigning the missing curing-protocol observations to a separate “protocol not recorded” category and re-running the corresponding curing-management model using all 3,106 records.

### 2.3 Definition and classification of scalding tobacco severity

The severity of scalding tobacco was classified according to the criteria specified in GB/T 23222–2008 *Classification and Investigation Methods for Tobacco Diseases and Insect Pests* and further verified using questionnaire survey records (Ren et al., 2008). Six severity levels were defined according to the proportion of affected leaf area: Level 0, no scalding tobacco; Level 1, scalding area ≤ 5% of total leaf area; Level 3, 6–10%; Level 5, 11–20%; Level 7, 21–40%; and Level 9, > 41% of the total leaf area. The numerical severity scores (0, 1, 3, 5, 7, and 9) were retained in subsequent severity-based analyses. For binary validation analyses, Levels 5, 7, and 9 were further grouped as severe scalding tobacco. Representative symptoms corresponding to different severity levels are shown in **Figure 2**.

**Figure 2:**
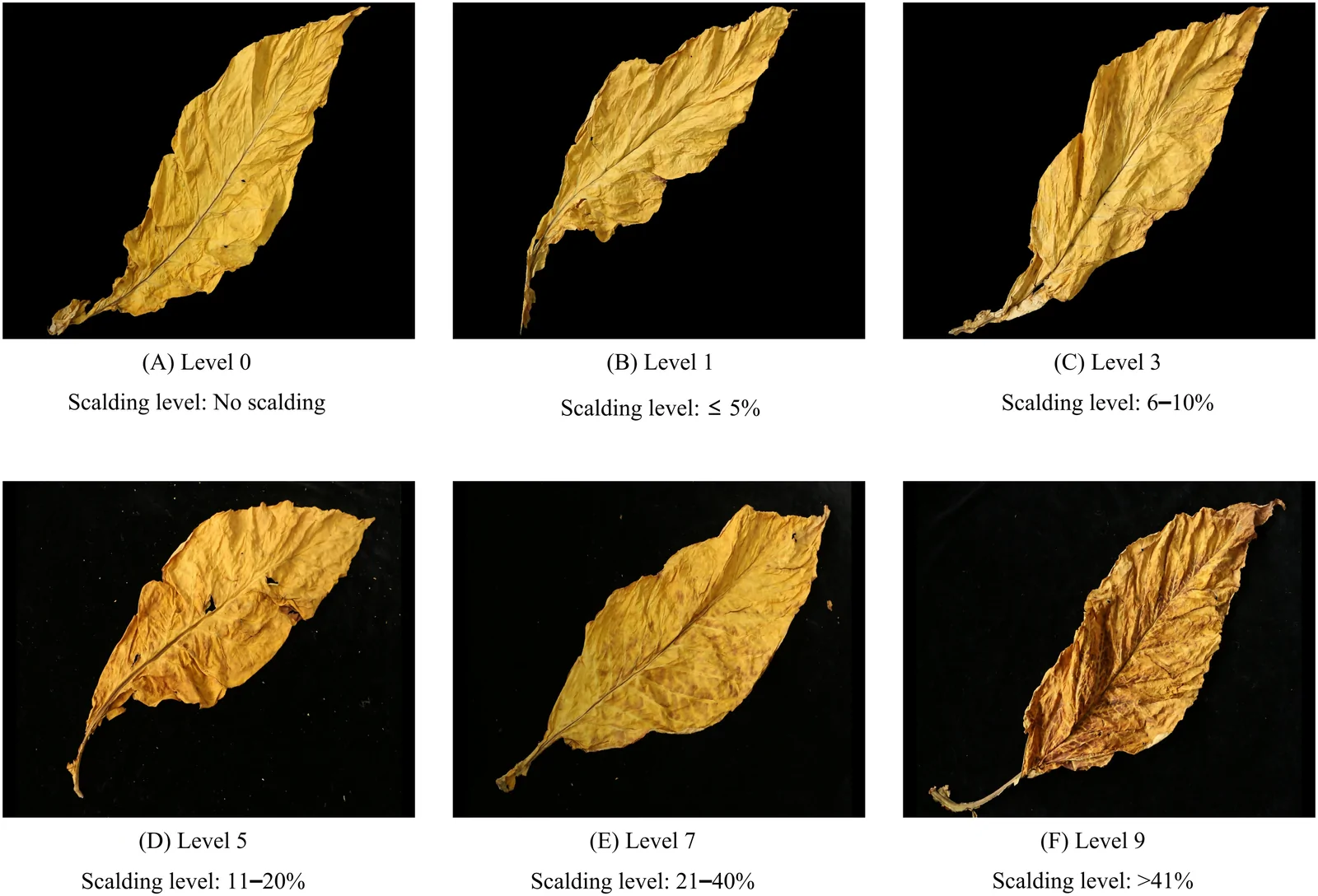
Representative symptoms of scalding tobacco leaves at different occurrence levels. **(A)**, Level 0 (no scalding); **(B)**, Level 1 (≤5%); **(C)**, Level 3 (6–10%); **(D)**, Level 5 (11–20%); **(E)**, Level 7 (21–40%); **(F)**, Level 9 (>41%). Levels were classified according to the proportion of affected leaf area.

### 2.4 Generation of spatial predictor variables and risk surfaces

To satisfy the input requirements of the MaxEnt model, all predictor variables were converted into raster layers prior to spatial modeling (Phillips et al., 2006; Elith et al., 2011). Spatial data processing was conducted separately for the climatic, field-management, and curing-management modules according to the corresponding modeling workflow. For the climatic-factor MaxEnt model, precipitation, annual mean temperature, and annual sunshine duration were converted into Arc/Info ASCII raster layers prior to model construction (Kass et al., 2021). Meteorological observations were available from 80 unique geographic locations. A regular longitude-latitude grid with a cell size of 0.02°was generated across the study area. For each climatic variable, linear interpolation was first applied to generate a continuous climatic surface, followed by nearest-neighbor interpolation to fill remaining grid cells without interpolated values. These interpolated layers were used to characterize regional-scale climatic gradients associated with scalding tobacco occurrence. Field-management and curing-management variables were derived from the questionnaire survey and represented management attributes observed at the sampling-point scale. Unlike the climatic predictors, management variables were not interpolated into continuous province-wide environmental raster layers before model fitting. Instead, each questionnaire record was linked directly to its corresponding geographic location, and the management attributes were prepared according to their variable types. For the field-management module, days after transplanting and potassium fertilizer application rate were represented as interval-based categorical predictors, whereas microbial inoculant application, potassium fertilizer management, potassium fertilizer type, and harvest maturity were standardized as categorical predictors. For the curing-management module, curing protocol was treated as a categorical predictor, whereas barn loading density was retained as a continuous numeric predictor. Categorical predictors were numerically encoded for model input; these numerical codes represented discrete management classes and therefore had no continuous quantitative interpretation. For the management modules, spatial risk surfaces were generated from model-predicted logistic outputs rather than by directly interpolating the original management variables. After 100 bootstrap model runs, the mean logistic output was calculated for each surveyed location, and these location-specific predictions were subsequently interpolated using IDW to generate continuous spatial risk surfaces (Matcham et al., 2021; Fung et al., 2022). The field-management risk surface was clipped to the administrative boundary of Yunnan Province, whereas the curing-management risk surface was clipped to the administrative boundaries of the prefecture-level regions represented in the occurrence dataset.

### 2.5 MaxEnt model development

Three module-specific occurrence-risk models were developed to evaluate climatic, field-management, and curing-management factors associated with scalding tobacco occurrence. For the climatic-factor module, the official MaxEnt Java software (version 3.4.4) was used (Phillips et al., 2006; Elith et al., 2011). Scalding tobacco occurrence records were used as response data, with interpolated raster layers of precipitation, annual mean temperature, and annual sunshine duration as environmental predictors. Logistic output was used to represent relative occurrence risk. The model was run with 100 bootstrap replicates, with 25% of occurrence records randomly withheld for testing. Model performance was evaluated using training and test AUC, and variable importance was assessed using percent contribution, permutation importance, jackknife tests, and response curves. The field-management and curing-management modules were analyzed using MaxEnt-style regularized logistic models. Level 0 was coded as non-occurrence (0), whereas Levels 1, 3, 5, 7, and 9 were coded as occurrence (1). The field-management model used 3,106 records and included interval-based categories of days after transplanting and potassium fertilizer application rate, together with microbial inoculant application, potassium fertilizer management, potassium fertilizer type, and harvest maturity; all predictors were treated as categorical variables and one-hot encoded. The curing-management model used 2,804 complete records, with curing protocol one-hot encoded as a categorical predictor and barn loading density standardized and entered as a continuous predictor. Both management models were evaluated using 100 bootstrap replicates, with out-of-bag observations used for testing. Model performance was assessed using training AUC, out-of-bag test AUC, and model gain. Variable importance was evaluated using relative contribution, permutation importance, and jackknife-style comparisons. Relative contribution was derived from the mean decrease in out-of-bag test AUC after predictor exclusion, whereas permutation importance was based on the corresponding AUC decrease after predictor permutation; both were normalized to sum to 100%. Jackknife-style comparisons included single-predictor and leave-one-predictor-out models, and response curves were used to characterize predictor–risk relationships.

### 2.6 Model evaluation and risk classification

Model performance was evaluated using the receiver operating characteristic (ROC) curve and the corresponding area under the curve (AUC) (Fielding and Bell, 1997; Phillips et al., 2006; Kass et al., 2021). For the climatic-factor MaxEnt model, AUC quantified discrimination between scalding tobacco occurrence records and background locations, whereas for the field-management and curing-management models, it quantified discrimination between occurrence and non-occurrence records. Because these modules differed in model structure, response representation, and evaluation framework, AUC values were interpreted within each module and were not directly compared among modules. Model stability was summarized using the mean ± standard deviation of training and test AUC across the 100 bootstrap replicates, with out-of-bag test AUC used for the management models. For the climatic-factor model, variable importance was evaluated using percent contribution, permutation importance, and jackknife tests generated by the official MaxEnt software. For the management models, variable importance was assessed using relative contribution, permutation importance, and jackknife-style comparisons. Relative contribution was calculated from the mean decrease in out-of-bag test AUC after predictor exclusion and normalized across predictors to sum to 100%. Permutation importance for the management models was similarly derived from the decrease in AUC after predictor permutation. Jackknife-style comparisons included single-predictor and leave-one-predictor-out models. The module-specific risk surfaces were subsequently classified using the Jenks natural breaks method (Jenks, 1967), which partitions continuous prediction values into homogeneous groups while maximizing differences among classes. The resulting risk surfaces were classified into four categories: low, moderate, high, and very high risk. These risk classes were used to characterize module-specific spatial patterns of predicted scalding tobacco occurrence risk.

### 2.7 Auxiliary statistical analysis

To further elucidate the effects of different factors on scalding tobacco severity, Random Forest (RF) models were developed and interpreted using Shapley Additive Explanations (SHAP) analysis (Breiman, 2001; Lundberg et al., 2017; Lundberg et al., 2020). Categorical predictors were one-hot encoded, and SHAP values for encoded categories were aggregated to the original predictor level. For the climatic-factor module, a weighted RF regression model was fitted to combination-level mean severity, with sample size used as weights, and a binary classification model for severe scalding was used for robustness evaluation. For the field-management module, a multiclass RF model was used as the primary analysis with grouped five-fold cross-validation, supplemented by binary classification and weighted regression models for robustness evaluation. For the curing-management module, a weighted RF regression model was used as the primary analysis, with binary classification for robustness evaluation. Missing curing-protocol records were additionally evaluated through a sensitivity analysis by treating them as a separate category. Classification performance was evaluated using overall accuracy, balanced accuracy, macro-F1 score, and ROC-AUC where applicable, whereas regression performance was assessed using RMSE, MAE, and R^2^. Predictor importance was quantified using permutation importance, and TreeSHAP was used to characterize the magnitude and direction of predictor contributions to model output (Fisher et al., 2019). The RF/SHAP analyses provided complementary severity-based evidence and were interpreted separately from the occurrence-risk models described above.

## 3 Results

### 3.1 Effects of different factors on the distribution of scalding tobacco severity levels

As shown in **Figure 3** and **Supplementary Table S2**, meteorological factors showed relatively limited differentiation in the distribution of scalding tobacco severity levels, whereas field-management and curing-stage management factors exerted more pronounced effects. Field-management factors significantly affected severity-level composition (*P* < 0.05). Microbial inoculant application increased the proportion of Level 5–9 leaves from 5.7% to 30.1%, indicating a shift from Level 3-dominated distribution toward higher severity levels. Potassium fertilizer application also markedly altered severity composition, increasing the proportion of Level 3–9 leaves from 12.7% in the non-application group to 89.3% in the application group. Among the surveyed potassium application-rate categories, the >450–675 kg ha^-1^ group was dominated by Level 5 (64.0%), whereas the >675 kg ha^-1^ group was entirely classified as Level 7. For days after transplanting, the 40–50 d group was entirely classified as Level 9, whereas within the surveyed 51–120 d range, severity generally shifted from Level 0 toward Level 7 with increasing days after transplanting. Harvest maturity also changed severity composition: mature leaves were mainly distributed in Level 0 and Level 1, early mature leaves were concentrated in Level 3 and Level 5, and over-mature leaves showed a higher proportion of Level 9. Curing-management factors also significantly influenced the distribution of scalding tobacco severity distribution (*P* < 0.05). The six-step three-stage curing process was dominated by Level 0 and Level 1 leaves and showed no Level 5–9 occurrence, whereas other curing processes exhibited higher proportions of Level 7 and Level 9, both reaching 14.1%. Loading capacity further altered severity composition. The lowest and highest loading-capacity groups showed relatively higher proportions of severe scalding, with Level 5–9 accounting for 22.8% in Q1 and 35.9% in Q4.

**Figure 3:**
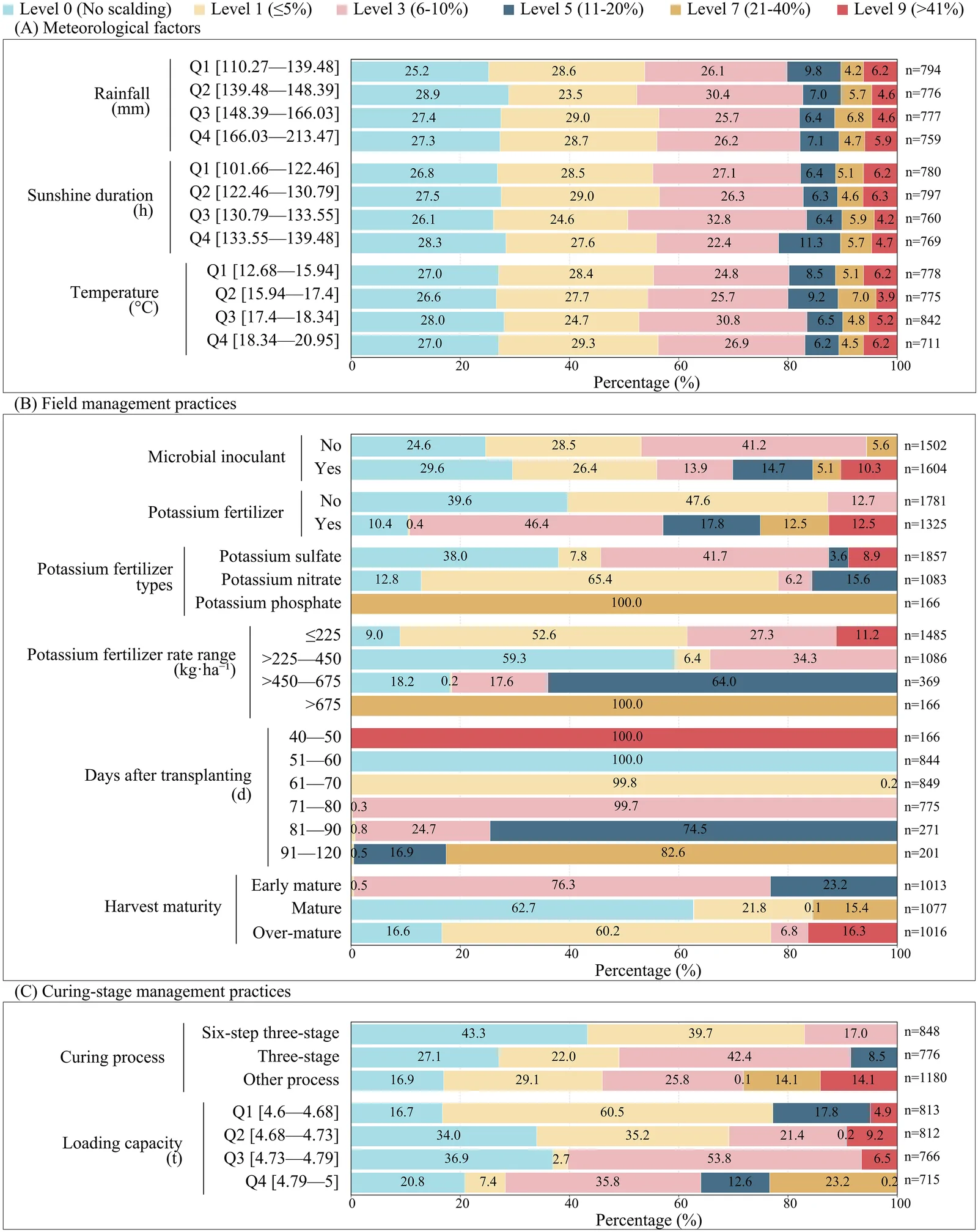
Distribution of scalding tobacco leaf occurrence levels under different influencing factors. The 100% stacked horizontal bars show the proportional composition of scalding tobacco leaf occurrence levels within each category or class of the corresponding influencing factor. Each horizontal bar represents one category, and different colors indicate six occurrence levels: Level 0, Level 1, Level 3, Level 5, Level 7, and Level 9. Values within bars represent the percentage of samples assigned to each occurrence level, and “n” on the right indicates the sample size for each category. Level 0 indicates no scalding; Level 1 indicates affected leaf area ≤5%; Level 3, 6–10%; Level 5, 11–20%; Level 7, 21–40%; and Level 9, >41%. **(A)** Meteorological factors, including rainfall, annual sunshine duration, and annual average temperature; continuous meteorological variables were classified into quartile-based groups (Q1–Q4). **(B)** Field management practices, including microbial inoculant application, potassium regulation, potassium fertilizer type, potassium application rate, days after transplanting for harvesting and curing, and tobacco leaf maturity. **(C)** Curing-stage management practices, including curing process and curing tobacco loading capacity. The x-axis represents the proportion of occurrence levels, and the stacked segments within each category sum to 100%.

### 3.2 Contributions, response relationships, and spatial effects of meteorological factors on scalding tobacco risk

The RF/SHAP analysis showed that precipitation had the largest mean absolute SHAP value among the three meteorological predictors, followed by annual mean temperature and annual sunshine duration (**Table 1**; **Supplementary Figure S1**). However, the meteorological-only RF severity regression model did not provide reliable out-of-sample predictive performance; accordingly, the RF/SHAP results were interpreted as exploratory model-attribution patterns rather than evidence for identifying a dominant meteorological predictor. The binary RF model also showed weak discrimination of severe scalding occurrence (ROC–AUC = 0.538; **Table 1**). In the spatial occurrence-risk analysis, the climatic MaxEnt model achieved mean training and testing AUC values of 0.7568 and 0.6831, respectively, with a testing AUC standard deviation of 0.0624, indicating moderate predictive performance and satisfactory model stability (**Supplementary Figure S3**). Percent contribution, permutation importance, and jackknife analyses identified annual sunshine duration as an important predictor of the spatial distribution of scalding tobacco occurrence risk (**Supplementary Table S3**). Annual sunshine duration also produced the highest training gain (0.1647) when used alone in the jackknife analysis (**Supplementary Figure S4**). Response curves showed nonlinear associations between climatic predictors and predicted occurrence risk. Precipitation exhibited a bimodal pattern, with relatively higher predicted risk at lower and higher precipitation levels and lower risk at intermediate levels, whereas annual sunshine duration and annual mean temperature showed interval-dependent changes in predicted risk (**Supplementary Figure S5**). Spatial predictions showed pronounced heterogeneity in scalding tobacco occurrence risk across Yunnan Province (**Figure 4**). Based on Jenks natural breaks classification, low-risk (Level I), moderate-risk (Level II), high-risk (Level III), and very high-risk (Level IV) zones accounted for 29.20%, 33.99%, 26.67%, and 10.13% of the study area, respectively, with high- and very high-risk zones together representing 36.80%. Very high-risk zones were mainly distributed from eastern Dali Prefecture to central-western Chuxiong Prefecture and extended into northwestern Yuxi and parts of central Yunnan, with smaller patches occurring in Zhaotong, Baoshan, Pu’er, and Honghe. High-risk zones formed continuous belts surrounding the very high-risk areas. Moderate-risk zones were widely distributed across parts of Zhaotong, Kunming, Qujing, and Lincang, whereas low-risk zones were primarily located in eastern Qujing, southwestern Lincang, and southeastern Honghe.

**Table 1:** Performance metrics and variable importance of the random forest model based on meteorological factors.

Item	Value
Number of survey records/unique climatic combinations	3,106/65
Weighted RMSE of severity regression model	0.382
Weighted R² of severity regression model	-0.228
Increase in RMSE after permutation of Prec/Temp/Sun	0.009/-0.001/-0.014
Mean absolute SHAP value of Prec/Temp/Sun	0.080/0.069/0.042
ROC–AUC of severe scalding tobacco classification model	0.538
Permutation importance of Temp/Prec/Sun in the binary classification model	0.033/0.008/0.008

Prec, precipitation; Temp, annual mean temperature; Sun, annual sunshine duration. RMSE, root mean square error; R^2^, coefficient of determination; SHAP, Shapley Additive Explanations; ROC–AUC, area under the receiver operating characteristic curve. The severity regression model was developed using the mean scalding tobacco severity index at the climatic-combination level, whereas the binary classification model was developed using severe scalding tobacco occurrence (Levels 5–9) as the response variable. Permutation importance was calculated based on the testing dataset.

**Figure 4:**
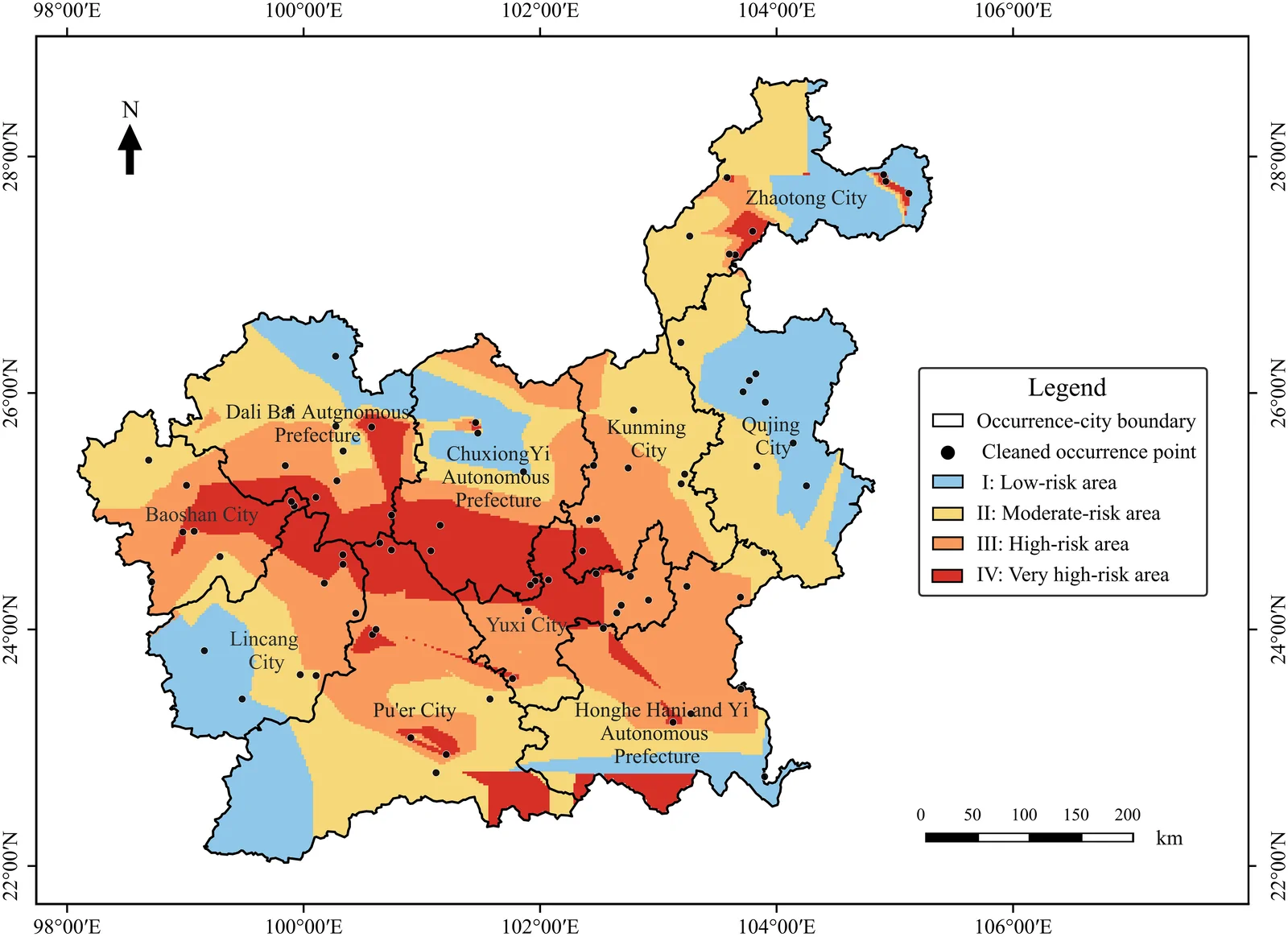
Spatial distribution of scalding tobacco risk based on meteorological factors.

### 3.3 Contributions, response relationships, and spatial effects of field-management practices on scalding tobacco risk

The RF/SHAP analyses identified days after transplanting as the most influential field-management predictor of scalding tobacco severity. Permutation importance analysis showed that permuting days after transplanting resulted in the largest decrease in balanced accuracy (0.457), exceeding the decreases associated with potassium fertilizer application rate (0.147) and the other field-management predictors (**Figure 5**; **Table 2**). SHAP analysis further showed that days after transplanting contributed across multiple severity levels, whereas potassium fertilizer application rate showed relatively greater contributions to moderate and severe scalding levels. In the spatial occurrence-risk analysis, days after transplanting also showed the largest contribution in the field-management model, with both relative contribution and permutation importance reaching 100.0% (**Supplementary Table S4**). Consistently, the jackknife analysis showed that the training gain obtained using days after transplanting alone (0.5797) was close to that of the full model (0.5809), while omission of this predictor reduced the training gain to 0.5511 (**Supplementary Figure S7**). The corresponding response curves showed that predicted occurrence risk varied primarily with days after transplanting (**Supplementary Figure S8**).

**Figure 5:**
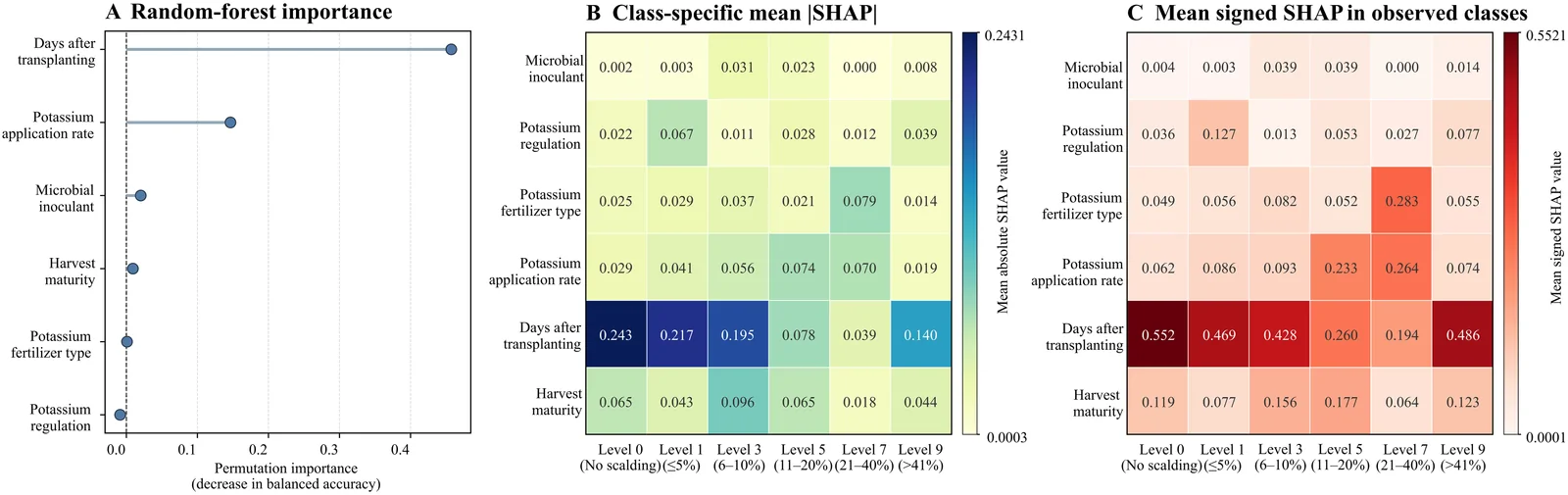
Random forest permutation importance and SHAP-based interpretation of field-management variables across scalding tobacco severity levels. **(A)** Permutation importance obtained from the six-class Random Forest model using grouped cross-validation. **(B)** Mean absolute SHAP values of field-management variables stratified by observed scalding tobacco severity level. Higher values indicate greater contributions of the corresponding predictors to model predictions. **(C)** Mean signed SHAP values of field-management variables stratified by observed scalding tobacco severity level, where positive and negative values denote positive and negative effects on predicted scalding tobacco severity, respectively.

**Table 2:** Performance metrics and variable importance of the random forest models based on field-management variables.

Item	Value
Number of records/unique management combinations (six-class model)	3,106/45
Cross-validated accuracy/balanced accuracy/macro-F1 (six-class model)	0.890/0.907/0.865
Permutation importance of days after transplanting/K rate	0.457/0.147
Mean	0.243/0.217/0.195
Signed SHAP value of K rate at Level 5/Level 7	0.233/0.264
Weighted R²/RMSE of robustness regression model	0.506/1.744
Balanced accuracy/ROC–AUC/F1 score of robustness binary classification model	0.711/0.930/0.540
Permutation importance of days after transplanting/potassium fertilizer management/K rate in the binary classification model	0.126/0.122/0.108

K rate, potassium fertilizer application rate; SHAP, Shapley Additive Explanations; RMSE, root mean square error; R^2^, coefficient of determination; ROC–AUC, area under the receiver operating characteristic curve. The six-class Random Forest model was developed using six scalding tobacco severity levels (Levels 0, 1, 3, 5, 7, and 9) as the response variable. Permutation importance values represent the reduction in model performance after randomly permuting a predictor. Mean |SHAP| values indicate the average magnitude of predictor contributions to model outputs, whereas signed SHAP values indicate the direction and magnitude of predictor effects. The robustness regression model was constructed using the mean scalding tobacco severity index at the management-combination level, while the robustness binary classification model was developed using severe scalding tobacco occurrence (Levels 5–9) as the response variable.

Spatial predictions based on field-management variables showed a clustered pattern, with the highest predicted occurrence risk concentrated mainly in Chuxiong Prefecture and surrounding areas (**Figure 6**). Low-risk (Level I), moderate-risk (Level II), high-risk (Level III), and very high-risk (Level IV) zones accounted for 0.59%, 44.83%, 51.79%, and 2.79% of the mapped area, respectively, with high- and very high-risk zones together accounting for 54.58%. Very high-risk zones were concentrated mainly in central-western and southern Chuxiong and extended into southeastern Dali, northwestern Yuxi, and parts of central Yunnan. Moderate-risk zones were distributed across parts of Zhaotong, Kunming, Yuxi, Pu’er, Lincang, and Baoshan, whereas low-risk zones occurred only as scattered patches.

**Figure 6:**
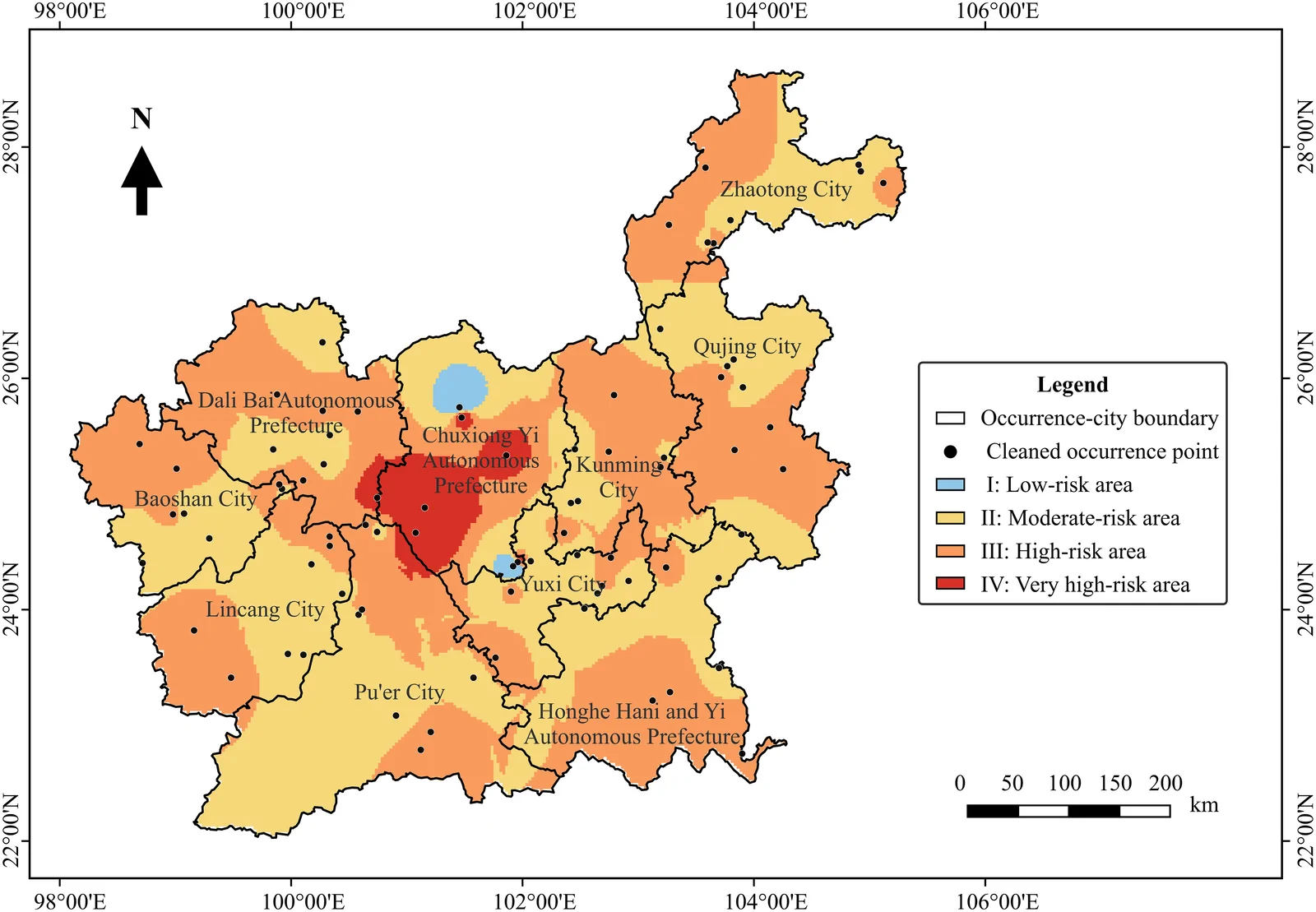
Predicted spatial distribution of scalding tobacco risk based on field-management variables.

### 3.4 Contributions, response relationships, and spatial effects of curing-management measures on scalding tobacco risk

The RF/SHAP analyses showed that barn loading density and curing protocol contributed differently to the prediction of scalding tobacco severity. In the severity regression model, permutation of barn loading density increased the weighted RMSE by 0.745, compared with 0.519 for curing protocol (**Figure 7**; **Table 3**). Permutation importance analysis showed that permutation of barn loading density increased the weighted RMSE by 0.745, exceeding the corresponding increase associated with curing protocol (0.519) (**Figure 7**; **Table 3**). SHAP analysis further revealed that barn loading density contributed more strongly to moderate and severe scalding levels, whereas curing protocol also contributed to the prediction of higher severity levels. The binary RF model for severe scalding achieved a balanced accuracy of 0.791, ROC–AUC of 0.879, and F1 score of 0.583, with permutation importance values of 0.196 and 0.181 for curing protocol and barn loading density, respectively. The sensitivity analysis incorporating the previously missing curing-protocol records yielded a similar ROC–AUC of 0.898.

**Figure 7:**
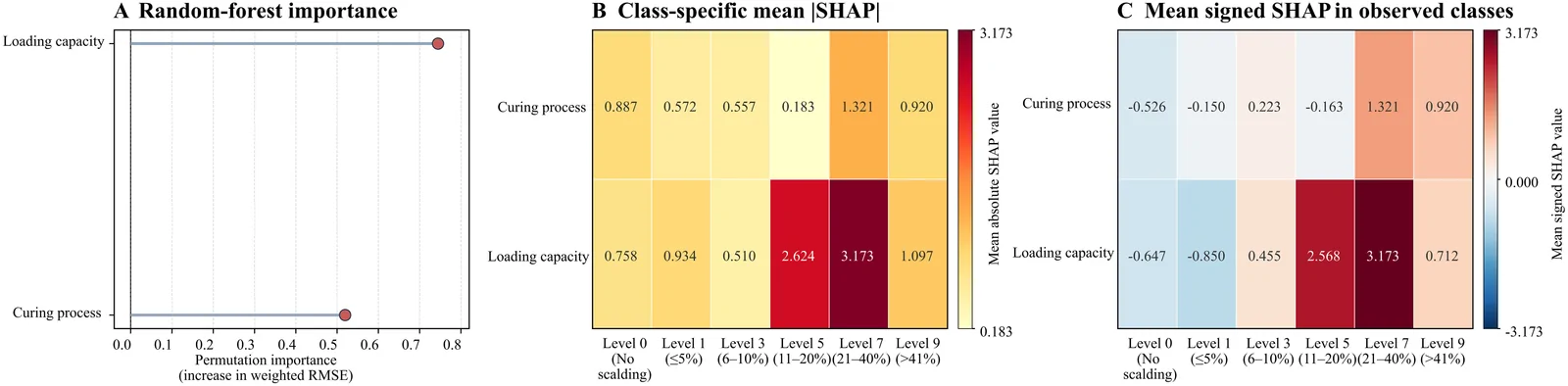
Random forest permutation importance and SHAP-based interpretation of curing-management variables. **(A)** Permutation importance derived from the Random Forest regression model based on the mean scalding tobacco severity index at the curing-management-combination level. **(B)** Mean absolute SHAP values of curing-management variables stratified by observed scalding tobacco severity levels, representing the magnitude of predictor contributions. **(C)** Mean signed SHAP values of curing-management variables stratified by observed scalding tobacco severity levels, where positive and negative values indicate positive and negative effects on predicted scalding tobacco severity, respectively.

**Table 3:** Performance metrics and variable importance of the random forest models based on curing-management variables.

Item	Value
Number of complete records/unique curing-management combinations	2,804/83
Weighted R^2^/RMSE/MAE of severity regression model	0.458/1.550/1.078
Increase in RMSE after permutation of barn loading density/curing protocol	0.745/0.519
Mean absolute SHAP value of barn loading density at Level 5/Level 7	2.624/3.173
Signed SHAP value of barn loading density at Level 5/Level 7	2.568/3.173
Balanced accuracy/ROC–AUC/F1 score of the primary binary classification model	0.791/0.879/0.583
Permutation importance of curing protocol/barn loading density in the primary binary classification model	0.196/0.181
Balanced accuracy/ROC–AUC of the sensitivity-analysis binary classification model (n = 3106)	0.779/0.898
Permutation importance of curing protocol/barn loading density in the sensitivity-analysis model	0.212/0.131

Results were obtained from Random Forest models and SHAP analyses based on curing-management variables. The primary analysis included 2804 records with complete curing-protocol and barn-loading-density information, representing 83 unique curing-management combinations. The mean scalding tobacco severity index was calculated for each combination. Model performance for severity regression was evaluated using R^2^, RMSE, and MAE. Permutation importance was quantified as the increase in RMSE after randomly permuting a predictor in the testing dataset, with larger values indicating greater predictive importance. Mean absolute SHAP values (|SHAP|) represent the average magnitude of predictor contributions, whereas signed SHAP values indicate the direction and magnitude of predictor effects. Level 5 and Level 7 correspond to scalding tobacco affecting 11–20% and 21–40% of the leaf area, respectively. In the severe-scolding classification model, Levels 5–9 were grouped as severe scalding tobacco, and model performance was assessed using balanced accuracy, ROC–AUC, and F1 score. Sensitivity analyses were conducted using all 3106 survey records, with missing curing-protocol observations assigned to a separate “protocol not recorded” category to evaluate the robustness of the primary results.

In the spatial occurrence-risk analysis, curing protocol showed the greater contribution in the curing-management model, with relative contribution and permutation importance values of 72.0% and 68.8%, respectively, compared with 28.0% and 31.2% for barn loading density (**Table 4**; **Supplementary Figure S9**). The jackknife analysis similarly showed that the training gain obtained using curing protocol alone (0.0739) approached that of the full model (0.0953) (**Supplementary Figure S10**). The corresponding response curves showed variation in predicted occurrence risk among curing protocols and a decreasing model-predicted risk with increasing barn loading density (**Supplementary Figure S11**). Spatial predictions based on curing-management variables showed relatively high predicted occurrence risk mainly in Dali Prefecture, Kunming City, and parts of central Yunnan, with higher-risk areas extending into Chuxiong, Qujing, Baoshan, and southern Honghe (**Figure 8**). Low-risk (Level I), moderate-risk (Level II), high-risk (Level III), and very high-risk (Level IV) zones accounted for 18.51%, 41.01%, 24.13%, and 16.35% of the mapped area, respectively, with high- and very high-risk zones together accounting for 40.48%. Very high-risk zones were concentrated mainly in central-eastern Dali, central-western Kunming, and parts of central Yunnan, whereas moderate- and low-risk zones occurred predominantly in the peripheral regions of the predicted high-risk areas.

**Table 4:** Percent contribution and permutation importance of curing-management variables in the MaxEnt model.

Variable	Percent contribution (%)	Permutation importance (%)
Curing protocol	72.0	68.8
Barn loading density (t)	28.0	31.2

**Figure 8:**
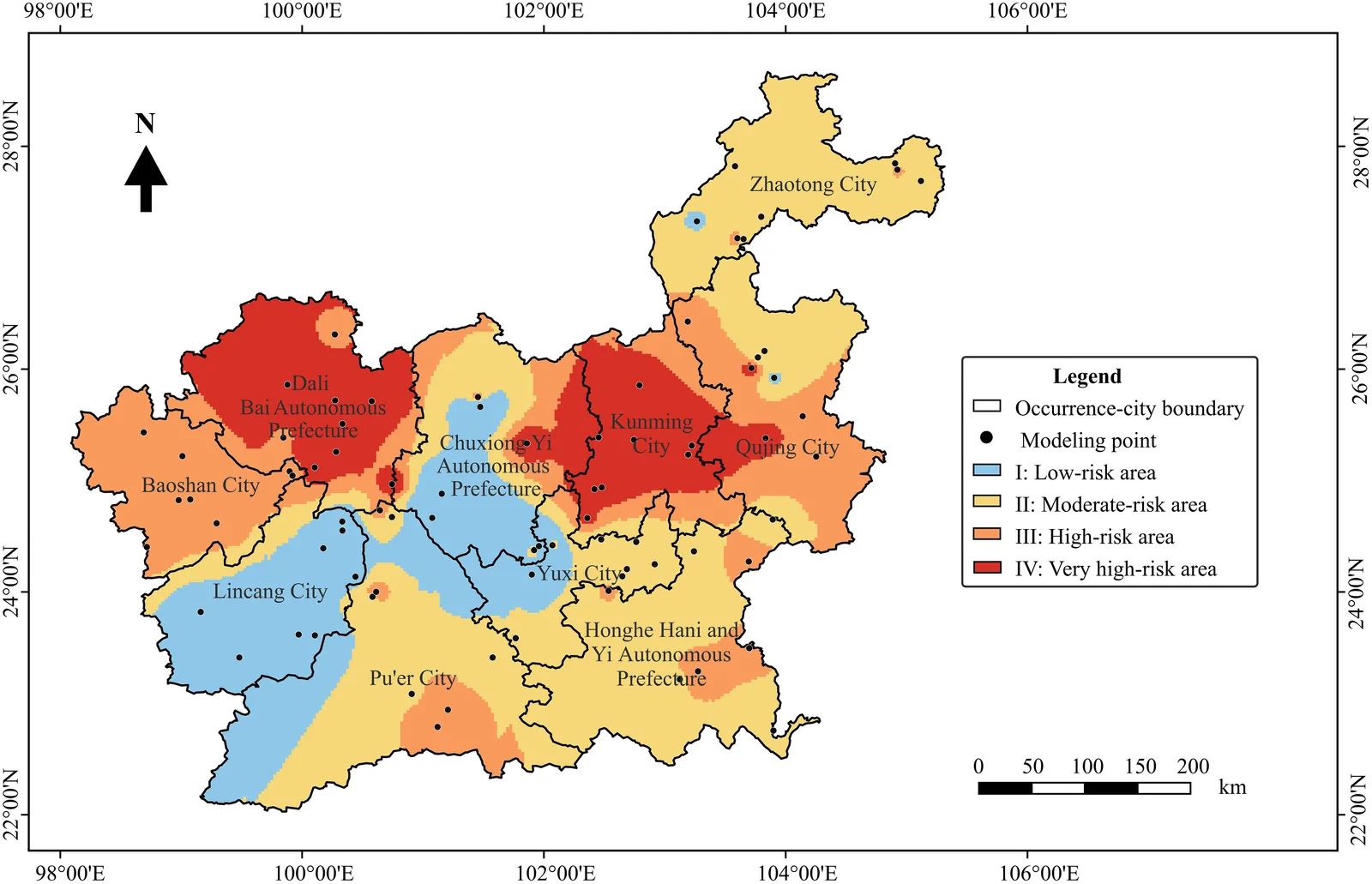
Predicted spatial distribution of scalding tobacco risk based on curing-management variables.

## 4 Discussion

### 4.1 Nonlinear climatic drivers and spatial heterogeneity of scalding tobacco risk

Climatic conditions influence not only tobacco growth and leaf maturation in the field but also the physiological processes associated with enzymatic browning, including the accumulation of phenolic substrates, the activities of oxidative enzymes such as polyphenol oxidase (PPO) and peroxidase (POD), leaf water status, and cell membrane integrity (Xu et al., 2025). The present study indicate that climatic conditions constitute an important environmental background for regional variation in scalding tobacco risk. However, the meteorological-only RF severity model did not provide reliable out-of-sample predictive performance, and the binary RF model also showed weak discrimination of severe scalding occurrence. Although precipitation received the largest SHAP attribution among the three meteorological predictors in the exploratory RF/SHAP analysis, this ranking should not be interpreted as evidence of a dominant independent effect on scalding severity. Precipitation, temperature, and sunshine duration may covary across the complex geographic and topographic gradients of Yunnan and contain partly shared predictive information.

In contrast, the spatial occurrence-risk analysis indicated that annual sunshine duration emerged as an important climatic predictor in the MaxEnt model and produced the highest training gain when used alone in the jackknife analysis. This result indicates that sunshine duration contained substantial information for characterizing the regional spatial pattern of scalding tobacco occurrence risk. Solar radiation can influence photosynthetic carbon assimilation, carbohydrate accumulation, leaf maturation, membrane stability, and antioxidant defense. Excessive irradiance may promote reactive oxygen species (ROS) accumulation and lipid peroxidation, potentially compromising membrane integrity and facilitating interactions between oxidative enzymes and phenolic substrates (Ciesielska et al., 2025). Conversely, insufficient radiation may limit photosynthate accumulation, delay leaf maturation, impair antioxidant capacity, thereby increasing susceptibility to browning-related disorders (Rong et al., 2025).

The MaxEnt response curves further revealed nonlinear associations between precipitation, annual sunshine duration, annual mean temperature, and predicted scalding tobacco occurrence risk. Precipitation displayed a bimodal response pattern, with relatively higher predicted risk at both lower and higher precipitation ranges, whereas sunshine duration and temperature showed interval-dependent responses. The nonlinear association with precipitation is physiologically plausible because water availability can influence leaf water status, tissue structure, maturation, and oxidative processes (Dong et al., 2023). Under suitable moisture conditions, tobacco plants maintain balanced water supply and carbon–nitrogen metabolism, resulting in coordinated leaf maturation and relatively intact cellular structures. In such conditions, phenolic substrates remain spatially separated from PPO and POD within cellular compartments, thereby reducing the likelihood of abnormal browning reactions (Ren et al., 2025). Conversely, excessive precipitation may lead to waterlogged soils, restricted root aeration, delayed leaf maturation, and elevated leaf moisture content at harvest (Liu et al., 2024). High tissue water content can prolong enzymatic activity during post-harvest handling and the early stages of curing, increasing the probability of phenolic oxidation mediated by PPO and POD. Consistent with this mechanism, Chen et al. (2024) reported that changes in precipitation significantly affected the activities of oxidative enzymes, including PPO, POD, and superoxide dismutase, in waxy yam, thereby altering browning intensity during post-harvest processing. These physiological mechanisms provide possible explanations for the observed precipitation-related patterns, although they were not directly measured in the present regional survey. The spatial predictions further revealed pronounced heterogeneity in climatic occurrence risk across Yunnan Province, with high-risk areas concentrated mainly in parts of central and western Yunnan. This spatial pattern suggests that different combinations of regional climatic conditions may create contrasting environmental backgrounds for scalding tobacco occurrence. Therefore, regional climatic conditions should be considered an important basis for spatially differentiated risk identification and monitoring, providing environmental context for the subsequent optimization of field and curing management practices.

### 4.2 Dominant regulatory effects of field-management practices on variations in scalding tobacco severity

Field-management practices were strongly associated with variations in scalding tobacco severity, with days after transplanting emerging as the most influential and consistently important predictor. Within the surveyed 51–120 d range, severity generally shifted toward higher levels with increasing days after transplanting, suggesting that harvest timing is closely associated with scalding susceptibility. Harvest timing is closely linked to leaf physiological maturity, and either premature or delayed harvesting may disrupt coordinated dehydration and membrane stability during curing, thereby increasing susceptibility to enzymatic browning (Zhao et al., 2020; Fan et al., 2026).

Potassium fertilizer application rate and potassium fertilizer management provided additional information on severity differentiation. The study demonstrated that potassium fertilization, together with fertilizer type and application rate, significantly altered the distribution of scalding tobacco severity levels, with higher potassium application rates generally associated with increased severity. Appropriate potassium supply is known to enhance osmotic regulation, maintain membrane stability, promote carbohydrate transport, and improve the coordination of leaf maturation. These effects contribute to reducing abnormal interactions between phenolic substrates and oxidative enzymes, thereby decreasing the likelihood of enzymatic browning (Sardans and Peñuelas, 2021). However, inappropriate fertilizer types, application methods, or excessive potassium inputs may disrupt nutrient balance and maturation processes, leading to altered leaf water status, carbon–nitrogen metabolism, and antioxidant capacity (Yu et al., 2026). In addition, Microbial inoculant application was also associated with differences in severity distribution, suggesting that biological-input management may represent an additional component of field-level scalding tobacco risk management. Microbial inoculants can influence nutrient availability and plant–soil interactions, thereby affecting crop growth and physiological performance (Shahwar et al., 2023; Yusuf et al., 2025). Recent evidence in tobacco further showed that microbial fertilizers can alter soil nutrient availability, enzymatic activity, and soil microbial and metabolic profiles, with corresponding effects on tobacco growth and development (2025). However, their specific role in scalding tobacco development requires further experimental validation. Collectively, these findings highlight the importance of harvest timing and coordinated nutrient management in reducing scalding tobacco risk. However, because days after transplanting was represented by interval-based categories, the present survey does not allow a precise optimal harvest window to be defined. Similarly, although relatively high severity was observed in the higher potassium application-rate categories, the >675 kg ha^-1^ category was open-ended and therefore should not be interpreted as establishing 675 kg ha^-1^ as a critical threshold. Therefore, field-management strategies are likely to be critical for reducing the occurrence of severe scalding tobacco and improving the quality of cured tobacco leaves.

### 4.3 Dominant regulatory effects of curing-management practices on scalding tobacco risk

Curing management represents an important post-harvest stage associated with the development of scalding tobacco because temperature, humidity, ventilation, and dehydration dynamics jointly determine the curing environment of harvested leaves. Appropriate coordination of yellowing, color fixing, and stem drying is essential for maintaining uniform moisture loss and color development during flue-curing. Recent studies have similarly shown that tobacco curing quality is sensitive to the control of temperature and humidity and to the spatial uniformity of the curing environment (Jia et al., 2023; Xiong et al., 2024). In this study, curing protocol showed greater importance for spatial occurrence-risk prediction, whereas barn loading density showed a stronger association with severity variation in the RF analysis. These results suggest that different components of curing management may be associated with different dimensions of scalding tobacco risk. Curing protocols determine the temporal sequence of temperature and humidity adjustment during yellowing, color fixing, and drying, and inappropriate stage transitions may result in poorly coordinated dehydration and color development. Previous studies have demonstrated that modification of curing temperature schedules can substantially affect the physical and chemical quality of flue-cured tobacco, supporting the importance of curing-process control for final leaf quality (Jia et al., 2023). Loading density can alter airflow resistance, heat transfer, moisture removal, and the spatial uniformity of temperature and humidity within curing barns. Experimental and modeling studies of bulk curing barns have shown that tobacco loading conditions influence internal temperature and humidity distributions and therefore need to be considered when adjusting curing parameters (Bai et al., 2017; Liu et al., 2022). More recent work has likewise shown that uneven temperature and humidity fields within curing chambers can adversely affect tobacco curing quality (Guo et al., 2025).

Spatial predictions based on curing-management variables showed relatively high predicted risk in Dali, Kunming, and parts of central Yunnan. However, these maps were generated from questionnaire-derived management records and spatial interpolation of model-predicted risk at surveyed locations. They therefore represent interpolated relative-risk patterns under the sampled curing-management combinations rather than actual continuous spatial distributions of curing practices. Uneven survey-point density, particularly in marginal areas, may further increase uncertainty in regions with limited spatial support. Therefore, future studies should combine more spatially balanced georeferenced management records, independent validation sites, objective monitoring of curing temperature, humidity, airflow, and loading conditions, and direct physiological measurements to improve both mechanistic interpretation and spatial transferability.

## 5 Conclusions

This study integrated severity analysis, RF modeling, SHAP interpretation, and MaxEnt modeling to evaluate the associations of meteorological factors, field-management practices, and curing-management measures with scalding tobacco risk in K326 flue-cured tobacco across the major tobacco-growing regions of Yunnan Province. Regional meteorological conditions provided an important basis for spatially differentiated risk identification, with higher occurrence risk concentrated mainly in parts of central and western Yunnan and annual sunshine duration showing the highest contribution in the meteorological MaxEnt model. Field-management practices showed strong associations with severity differentiation, with days after transplanting emerging as the most influential predictor, while curing-management practices represented an additional key management dimension associated with scalding tobacco occurrence and severity. Collectively, these findings support a shift from curing-centered control toward an integrated management strategy combining meteorological risk identification, appropriate harvest timing, coordinated nutrient management, and precision curing. However, the observational design, regional and cultivar specificity, questionnaire-derived management information, and lack of direct physiological and biochemical measurements limit the broader generalization and mechanistic interpretation of these findings. Future studies should incorporate multi-year, multi-region, and multi-cultivar validation, together with more finely resolved management experiments and direct physiological and biochemical measurements.

## Data Availability

The original contributions presented in the study are included in the article/**Supplementary Material**. Further inquiries can be directed to the corresponding authors.
