# Supplementary material for: Spatial distribution and driving factors of scalding tobacco risk in K326 flue-cured tobacco in Yunnan Province, China
Source: Front Plant Sci. 2026 Sep 22;17:1922535. doi: 10.3389/fpls.2026.1922535 (PMC13640454; doi:10.3389/fpls.2026.1922535)
Supplement: Supplementary file 1 [file SupplementaryFile1.docx]

**Supplementary Tables and Figures**

# Tables

## **Supplementary Table 1. The questionnaire used to investigate factors associated with scalding tobacco leaves after curing**

**General information recorded before the questionnaire:**

Prefecture/city: ____________________; county/city: ____________________; township: ____________________.

Village committee/street/community: ____________________; planted tobacco cultivar: K326.

| **No.** | **Survey item** | **Response options / information requested** | **Response format** |
| --- | --- | --- | --- |
| 1 | Type of potassium fertilizer applied in the tobacco field | A. Potassium sulfate B. Potassium phosphate C. Potassium nitrate | Multiple choice |
| 2 | Total potassium fertilizer application rate | Record the amount applied (kg mu^-1^) | Open-ended |
| 3 | Potassium fertilizer application method | Record the application method | Open-ended |
| 4 | Days after transplanting when the harvested tobacco leaves were collected for curing | Record the number of days after transplanting | Open-ended |
| 5 | Maturity and yellowing degree of the harvested tobacco leaves | Describe the maturity and yellowing status | Open-ended |
| 6 | Loading capacity of the curing barn | Record the loading capacity | Open-ended |
| 7 | Occurrence of scalding tobacco leaves after curing | A. None B. <5% C. 6–10% D. 11–20% E. 21–40% F. >41% | Multiple choice |
| 8 | Curing protocol used for the harvested tobacco leaves | Photograph the protocol and provide it to the investigator | Photo record |
| 9 | Primary cause of scalding tobacco leaves attributable to fresh-leaf quality | A. Field waterlogging B. Fertilization C. Low temperature/cold injury D. Micronutrient toxicity | Multiple choice |
| 10 | Primary cause of scalding tobacco leaves during curing | A. Fresh-leaf quality B. Fire-management technique C. Curing protocol D. Other causes (please specify) | Multiple choice |
| 11 | Primary fire-management cause of scalding tobacco leaves at your location | A. Operator absent from duty B. Substandard fuel C. Irregular coal/fuel feeding D. Other causes (please specify) | Multiple choice |
| 12 | Primary curing-protocol cause of ash-grey tobacco leaves at your location | A. Wet-bulb temperature set too high B. Yellowing stage too long C. Temperature increase too rapid during the color-fixing stage D. Other causes (please specify) | Multiple choice |
| 13 | Other main causes of scalding tobacco leaves at your location | A. No B. Yes (please specify) | Multiple choice with specification |
| 14 | Use of microbial inoculants for prevention/control in fields prone to scalding tobacco leaves | A. No B. Yes (please specify) | Multiple choice with specification |
| 15 | Use of potassium fertilizer regulation/control measures in fields prone to scalding tobacco leaves | A. No B. Yes (please specify) | Multiple choice with specification |
| 16 | Methods used to solve or control the scalding tobacco leaf problem | Describe the measures used | Open-ended |

Note: 1 mu = 666.7 m^2^. “scalding tobacco leaves” refers to cured tobacco leaves showing greyish or ash-like discoloration after curing.

*Response-format wording was standardized to distinguish pure multiple-choice questions, open-ended entries, photo records, and multiple-choice questions requiring additional specification when “Yes/Other” was selected.*

## **Supplementary Table 2. Distribution and significance groups of scalding tobacco leaf occurrence levels under different influencing factors**

| **Influencing factor** | **Level** | **n** | **Level 0** | **Level 1** | **Level 3** | **Level 5** | **Level 7** | **Level 9** |
| --- | --- | --- | --- | --- | --- | --- | --- | --- |
| Rainfall (mm) | Q1 [110.27, 139.48] | 794 | 25.2 ± 1.5 Aa | 28.6 ± 1.6 Aa | 26.1 ± 1.6 Aac | 9.8 ± 1.1 Ba | 4.2 ± 0.7 Cbcd | 6.2 ± 0.9 Ca |
| Rainfall (mm) | Q2 [139.48, 148.39] | 776 | 28.9 ± 1.6 Aa | 23.5 ± 1.5 Bb | 30.4 ± 1.7 Aa | 7.0 ± 0.9 Cbc | 5.7 ± 0.8 Cac | 4.6 ± 0.8 Ca |
| Rainfall (mm) | Q3 [148.39, 166.03] | 777 | 27.4 ± 1.6 Aa | 29.0 ± 1.6 Aa | 25.7 ± 1.6 Abcd | 6.4 ± 0.9 Bbd | 6.8 ± 0.9 Ba | 4.6 ± 0.8 Ba |
| Rainfall (mm) | Q4 [166.03, 213.47] | 759 | 27.3 ± 1.6 Aa | 28.7 ± 1.6 Aa | 26.2 ± 1.6 Aad | 7.1 ± 0.9 Bacd | 4.7 ± 0.8 Bad | 5.9 ± 0.9 Ba |
| Sunshine duration (h) | Q1 [101.66, 123.46] | 780 | 26.8 ± 1.6 Aa | 28.5 ± 1.6 Aa | 27.1 ± 1.6 Ab | 6.4 ± 0.9 Bb | 5.1 ± 0.8 Ba | 6.2 ± 0.9 Ba |
| Sunshine duration (h) | Q2 [123.46, 130.79] | 797 | 27.5 ± 1.6 Aa | 29.0 ± 1.6 Aa | 26.3 ± 1.6 Abd | 6.3 ± 0.9 Bb | 4.6 ± 0.7 Ba | 6.3 ± 0.9 Ba |
| Sunshine duration (h) | Q3 [130.79, 133.55] | 760 | 26.1 ± 1.6 Ba | 24.6 ± 1.6 Ba | 32.8 ± 1.7 Aa | 6.4 ± 0.9 Cb | 5.9 ± 0.9 Ca | 4.2 ± 0.7 Ca |
| Sunshine duration (h) | Q4 [133.55, 139.48] | 769 | 28.3 ± 1.6 Aa | 27.6 ± 1.6 Aa | 22.4 ± 1.5 Bcd | 11.3 ± 1.1 Ca | 5.7 ± 0.8 Da | 4.7 ± 0.8 Da |
| Temperature (℃) | Q1 [12.68, 16.08] | 778 | 27.0 ± 1.6 Aa | 28.4 ± 1.6 Aac | 24.8 ± 1.5 Abc | 8.5 ± 1.0 Bacd | 5.1 ± 0.8 CDac | 6.2 ± 0.9 BDa |
| Temperature (℃) | Q2 [16.08, 17.38] | 775 | 26.6 ± 1.6 Aa | 27.7 ± 1.6 Aad | 25.7 ± 1.6 Abd | 9.2 ± 1.0 Ba | 7.0 ± 0.9 Ba | 3.9 ± 0.7 Cbc |
| Temperature (℃) | Q3 [17.38, 18.34] | 842 | 28.0 ± 1.5 ADa | 24.7 ± 1.5 BDbcd | 30.8 ± 1.6 Aa | 6.5 ± 0.9 Cbc | 4.8 ± 0.7 Cad | 5.2 ± 0.8 Cac |
| Temperature (℃) | Q4 [18.34, 20.95] | 711 | 27.0 ± 1.7 Aa | 29.3 ± 1.7 Aa | 26.9 ± 1.7 Aacd | 6.2 ± 0.9 Bbd | 4.5 ± 0.8 Bbcd | 6.2 ± 0.9 Ba |
| Microbial inoculant application | No | 166 | 0.0 ± 0.0 Bb | 0.0 ± 0.0 Bbd | 0.0 ± 0.0 Bc | 0.0 ± 0.0 Bc | 0.0 ± 0.0 Bb | 100.0 ± 0.0 Aa |
| Microbial inoculant application | Yes | 844 | 100.0 ± 0.0 Aa | 0.0 ± 0.0 Bcde | 0.0 ± 0.0 Bc | 0.0 ± 0.0 Bc | 0.0 ± 0.0 Bb | 0.0 ± 0.0 Bb |
| Potassium fertilizer application | No | 849 | 0.0 ± 0.0 Bb | 99.8 ± 0.2 Aa | 0.2 ± 0.2 Bc | 0.0 ± 0.0 Bc | 0.0 ± 0.0 Bb | 0.0 ± 0.0 Bb |
| Potassium fertilizer application | Yes | 775 | 0.0 ± 0.0 Bb | 0.3 ± 0.2 Bbe | 99.7 ± 0.2 Aa | 0.0 ± 0.0 Bc | 0.0 ± 0.0 Bb | 0.0 ± 0.0 Bb |
| Potassium fertilizer application types | Potassium sulfate | 271 | 0.0 ± 0.0 Cb | 0.7 ± 0.5 Cb | 24.7 ± 2.6 Bb | 74.5 ± 2.6 Aa | 0.0 ± 0.0 Cb | 0.0 ± 0.0 Cb |
| Potassium fertilizer application types | Potassium nitrate | 201 | 0.0 ± 0.0 Cb | 0.5 ± 0.5 Cb | 0.0 ± 0.0 Cc | 16.9 ± 2.6 Bb | 82.6 ± 2.7 Aa | 0.0 ± 0.0 Cb |
| Potassium fertilizer application types | Potassium phosphate | 1013 | 0.0 ± 0.0 Dc | 0.5 ± 0.2 Cc | 76.3 ± 1.3 Aa | 23.2 ± 1.3 Ba | 0.0 ± 0.0 Db | 0.0 ± 0.0 Db |
| Potassium fertilizer application rate range (kg ha^-1^) | ≤225 | 1077 | 62.7 ± 1.5 Aa | 21.8 ± 1.3 Bb | 0.0 ± 0.0 Dc | 0.1 ± 0.1 Db | 15.4 ± 1.1 Ca | 0.0 ± 0.0 Db |
| Potassium fertilizer application rate range (kg ha^-1^) | 225–450 | 1016 | 16.6 ± 1.2 Bb | 60.2 ± 1.5 Aa | 6.8 ± 0.8 Cb | 0.0 ± 0.0 Db | 0.0 ± 0.0 Db | 16.3 ± 1.2 Ba |
| Potassium fertilizer application rate range (kg ha^-1^) | 450–675 | 1502 | 24.6 ± 1.1 Cb | 28.5 ± 1.2 Ba | 41.2 ± 1.3 Aa | 0.1 ± 0.1 Eb | 5.6 ± 0.6 Da | 0.0 ± 0.0 Eb |
| Potassium fertilizer application rate range (kg ha^-1^) | >675 | 1604 | 29.6 ± 1.1 Aa | 26.4 ± 1.1 Ba | 13.9 ± 0.9 Cb | 14.7 ± 0.9 Ca | 5.1 ± 0.5 Ea | 10.3 ± 0.8 Da |
| Days after transplanting for harvesting and curing (d) | 40-50 | 1781 | 39.6 ± 1.2 Ba | 47.6 ± 1.2 Aa | 12.7 ± 0.8 Cb | 0.0 ± 0.0 Db | 0.0 ± 0.0 Db | 0.0 ± 0.0 Db |
| Days after transplanting for harvesting and curing (d) | 51-60 | 1325 | 10.4 ± 0.8 Cb | 0.3 ± 0.2 Db | 46.4 ± 1.4 Aa | 17.8 ± 1.1 Ba | 12.5 ± 0.9 Ca | 12.5 ± 0.9 Ca |
| Days after transplanting for harvesting and curing (d) | 61-70 | 1485 | 9.0 ± 0.7 Dc | 52.6 ± 1.3 Aa | 27.3 ± 1.2 Bb | 0.0 ± 0.0 Eb | 0.0 ± 0.0 Eb | 11.2 ± 0.8 Ca |
| Days after transplanting for harvesting and curing (d) | 71-80 | 1086 | 59.3 ± 1.5 Aa | 6.4 ± 0.7 Cb | 34.3 ± 1.4 Ba | 0.0 ± 0.0 Db | 0.0 ± 0.0 Db | 0.0 ± 0.0 Db |
| Days after transplanting for harvesting and curing (d) | 81-90 | 369 | 18.2 ± 2.0 Bb | 0.3 ± 0.3 Cc | 17.6 ± 2.0 Bc | 64.0 ± 2.5 Aa | 0.0 ± 0.0 Cb | 0.0 ± 0.0 Cb |
| Days after transplanting for harvesting and curing (d) | 91-120 | 166 | 0.0 ± 0.0 Bd | 0.0 ± 0.0 Bc | 0.0 ± 0.0 Bd | 0.0 ± 0.0 Bb | 100.0 ± 0.0 Aa | 0.0 ± 0.0 Bb |
| Harvest maturity | Early mature | 1857 | 38.0 ± 1.1 Ba | 7.8 ± 0.6 Cb | 41.7 ± 1.1 Aa | 3.6 ± 0.4 Db | 0.0 ± 0.0 Eb | 8.9 ± 0.7 Ca |
| Harvest maturity | Mature | 1083 | 12.8 ± 1.0 Bb | 65.4 ± 1.4 Aa | 6.2 ± 0.7 Cb | 15.6 ± 1.1 Ba | 0.0 ± 0.0 Db | 0.0 ± 0.0 Db |
| Harvest maturity | Over-mature | 166 | 0.0 ± 0.0 Bc | 0.0 ± 0.0 Bc | 0.0 ± 0.0 Bc | 0.0 ± 0.0 Bc | 100.0 ± 0.0 Aa | 0.0 ± 0.0 Bb |
| Curing process | Six-step three-stage | 848 | 43.3 ± 1.7 Aa | 39.7 ± 1.7 Aa | 17.0 ± 1.3 Bc | 0.0 ± 0.0 Cb | 0.0 ± 0.0 Cb | 0.0 ± 0.0 Cb |
| Curing process | Three-stage | 776 | 27.1 ± 1.6 Bb | 22.0 ± 1.5 Cc | 42.4 ± 1.8 Aa | 8.5 ± 1.0 Da | 0.0 ± 0.0 Eb | 0.0 ± 0.0 Eb |
| Curing process | Other process | 1180 | 16.9 ± 1.1 Bc | 29.1 ± 1.3 Ab | 25.8 ± 1.3 Ab | 0.1 ± 0.1 Cb | 14.1 ± 1.0 Ba | 14.1 ± 1.0 Ba |
| Loading capacity (t) | Q1 [4.6, 4.69] | 813 | 16.7 ± 1.3 Bc | 60.5 ± 1.7 Aa | 0.0 ± 0.0 Dd | 17.8 ± 1.3 Ba | 0.0 ± 0.0 Db | 4.9 ± 0.8 Cb |
| Loading capacity (t) | Q2 [4.69, 4.73] | 812 | 34.0 ± 1.7 Aa | 35.2 ± 1.7 Ab | 21.4 ± 1.4 Bc | 0.1 ± 0.1 Dc | 0.0 ± 0.0 Db | 9.2 ± 1.0 Ca |
| Loading capacity (t) | Q3 [4.73, 4.78] | 766 | 36.9 ± 1.7 Ba | 2.7 ± 0.6 Dd | 53.8 ± 1.8 Aa | 0.0 ± 0.0 Ec | 0.0 ± 0.0 Eb | 6.5 ± 0.9 Cb |
| Loading capacity (t) | Q4 [4.78, 5] | 715 | 20.8 ± 1.5 Bb | 7.4 ± 1.0 Dc | 35.8 ± 1.8 Ab | 12.6 ± 1.2 Cb | 23.2 ± 1.6 Ba | 0.1 ± 0.1 Ec |

Note: Values are shown as occurrence-level proportion (%) ± SE followed by significance letters. The proportion represents the percentage of samples assigned to each scalding tobacco leaf occurrence level within the corresponding category of each influencing factor. SE was calculated as sqrt[p(1-p)/n] × 100, where p is the occurrence-level proportion expressed as a decimal and n is the number of observations in that category. Occurrence levels follow the grading scheme used in the original survey and composite bar figure: Level 0 = no scalding; Level 1 = affected leaf area <5%; Level 3 = 6–10%; Level 5 = 11–20%; Level 7 = 21–40%; and Level 9 = >41%. Uppercase letters compare occurrence levels within the same row/category, whereas lowercase letters compare the same occurrence level among categories within the same panel; values sharing at least one letter are not significantly different at *P < 0.05*. Q1–Q4 indicate quartile-based classes for continuous variables; intervals are shown exactly as used for the analysis. The n column gives the sample size for each category, and therefore n is not repeated in the Level column.

## **Supplementary Table 3.** Percent contribution and permutation importance of climatic variables in the MaxEnt model

| **Variable** | **Percent contribution (%)** | **Permutation importance (%)** |
| --- | --- | --- |
| Prec | 29.6 | 21.2 |
| Sun | 48.7 | 55.5 |
| Temp | 21.7 | 23.4 |

Note: Prec, precipitation; Sun, annual sunshine duration; Temp, annual mean temperature. Percent contribution represents the relative contribution of each predictor during model training and reflects its influence on model gain. Permutation importance was calculated by randomly permuting the values of each predictor and measuring the resulting decrease in model performance; higher values indicate greater independent predictive information and lower redundancy with other variables. Both metrics were generated by MaxEnt version 3.4.4 and were used to evaluate the relative importance of climatic variables in explaining the spatial distribution of scalding tobacco risk in Yunnan Province.

## **Supplementary Table 4.** Relative contribution and permutation importance of Field-Management variables in the MaxEnt model

| **Variable** | **Relative contribution (%)** | **Permutation importance (%)** |
| --- | --- | --- |
| Days from transplanting to harvesting (DTH) | 100.0 | 100.0 |
| Potassium fertilizer application rate (K rate, kg ha^-1^) | 0.0 | 0.0 |
| Microbial inoculant application | 0.0 | 0.0 |
| Harvest maturity | 0.0 | 0.0 |
| Potassium fertilizer type | 0.0 | 0.0 |
| Potassium fertilizer management | 0.0 | 0.0 |

Note: DTH, days from transplanting to harvesting; K rate, potassium fertilizer application rate. Relative contribution and permutation importance were derived from decreases in out-of-bag test AUC following predictor exclusion and permutation, respectively, and were normalized across predictors to sum to 100% (see Section 2.5 for details).

# Figures

## **Supplementary Figure 1.**

Exploratory variable-attribution patterns from the climatic Random Forest severity model. (A) Permutation importance obtained from the Random Forest regression model developed using the mean scalding tobacco severity index at the climatic-combination level. The x-axis indicates the mean increase in weighted RMSE following permutation of each predictor; negative values indicate no consistent contribution to out-of-sample prediction. (B) Mean absolute SHAP values of climatic predictors stratified by observed scalding tobacco severity level, representing the magnitude of predictor contributions. (C) Mean signed SHAP values across severity levels, with positive and negative values indicating contributions toward increasing and decreasing the corresponding model output, respectively.

## **Supplementary Figure 2.**

SHAP value distributions of climatic predictors across six scalding tobacco severity levels. Panels A–F correspond to Level 0, Level 1, Level 3, Level 5, Level 7, and Level 9, respectively. The horizontal axis represents the SHAP value for the corresponding severity class, with positive and negative values indicating contributions toward increasing and decreasing the model output for that class, respectively. Colors represent the relative values of each meteorological variable after standardization on the independent test set, ranging from blue (low) to red (high).

## **Supplementary Figure 3.**

AUC evaluation of the Climatic-Factor model

## **Supplementary Figure 4.**

Jackknife test of the Climatic-Factor MaxEnt model,

## **Supplementary Figure 5.**

Response curves of climatic variables in the Climatic-Factor MaxEnt model.

## **Supplementary Figure 6.**

AUC assessment of the Field-Management MaxEnt model.

## **Supplementary Figure 7.**

Jackknife test of the Field-Management MaxEnt model.

## Supplementary Figure 8.

Response curves of the Field-Management MaxEnt model.

## **Supplementary Figure 9.**

AUC assessment of the curing-management maxent model.

## **Supplementary Figure 10.**

Jackknife analysis of variable importance in the curing-management maxent model.

## **Supplementary Figure 11.**

Response curves of Curing-Management variables in the Curing-Management MaxEnt.
